# Patient-Provider Interactions Affect Symptoms in Gastroesophageal Reflux Disease: A Pilot Randomized, Double-Blind, Placebo-Controlled Trial

**DOI:** 10.1371/journal.pone.0136855

**Published:** 2015-09-30

**Authors:** Michelle L. Dossett, Lin Mu, Roger B. Davis, Iris R. Bell, Anthony J. Lembo, Ted J. Kaptchuk, Gloria Y. Yeh

**Affiliations:** 1 Department of Medicine, Division of General Medicine and Primary Care, Beth Israel Deaconess Medical Center, Boston, Massachusetts, United States of America; 2 Clinical Research Center, Beth Israel Deaconess Medical Center, Boston, Massachusetts, United States of America; 3 Department of Biostatistics, Harvard T. H. Chan School of Public Health, Boston, Massachusetts, United States of America; 4 Department of Family and Community Medicine, The University of Arizona College of Medicine, Tucson, Arizona, United States of America; 5 Department of Medicine, Division of Gastroenterology, Beth Israel Deaconess Medical Center, Boston, Massachusetts, United States of America; 6 Program in Placebo Studies, Beth Israel Deaconess Medical Center and Harvard Medical School, Boston, Massachusetts, United States of America; University Hospital Llandough, UNITED KINGDOM

## Abstract

**Background:**

It is unclear whether the benefits that some patients derive from complementary and integrative medicine (CIM) are related to the therapies recommended or to the consultation process as some CIM provider visits are more involved than conventional medical visits. Many patients with gastrointestinal conditions seek out CIM therapies, and prior work has demonstrated that the quality of the patient-provider interaction can improve health outcomes in irritable bowel syndrome, however, the impact of this interaction on gastroesophageal reflux disease (GERD) is unknown. We aimed to assess the safety and feasibility of conducting a 2x2 factorial design study preliminarily exploring the impact of the patient-provider interaction, and the effect of an over-the-counter homeopathic product, Acidil, on symptoms and health-related quality of life in subjects with GERD.

**Methods:**

24 subjects with GERD-related symptoms were randomized in a 2x2 factorial design to receive 1) either a standard visit based on an empathic conventional primary care evaluation or an expanded visit with questions modeled after a CIM consultation and 2) either Acidil or placebo for two weeks. Subjects completed a daily GERD symptom diary and additional measures of symptom severity and health-related quality of life.

**Results:**

There was no significant difference in GERD symptom severity between the Acidil and placebo groups from baseline to follow-up (p = 0.41), however, subjects who received the expanded visit were significantly more likely to report a 50% or greater improvement in symptom severity compared to subjects who received the standard visit (p = 0.01). Total consultation length, perceived empathy, and baseline beliefs in CIM were not associated with treatment outcomes.

**Conclusion:**

An expanded patient-provider visit resulted in greater GERD symptom improvement than a standard empathic medical visit. CIM consultations may have enhanced placebo effects, and further studies to assess the active components of this visit-based intervention are warranted.

**Trial Registration:**

ClinicalTrials.gov NCT01915173

## Introduction

Studies in the conventional medical setting have demonstrated that the patient-provider interaction can affect response to treatment [1–3]. In irritable bowel syndrome, the quality of the therapeutic encounter significantly affects response rates to placebo treatment [4]. Notably, patients’ experiences of the therapeutic relationship in the context of visits to complementary and integrative medicine (CIM) providers is often quite different than their experiences in conventional medical settings [5–9]. While differences in visit lengths may be a contributing factor, the unique questions included in some CIM providers’ histories may alter patients’ perspectives of their symptoms, thereby promoting coping and symptom improvement [5,10]. Consequently, some patients may improve clinically as a result of the enhanced placebo effects of the CIM consultation process itself, irrespective of the therapy offered [8].

Gastroesphageal reflux disease (GERD) is one of the most prevalent health-related conditions in the Western world with prevalence estimates ranging from 20–40% [11,12]. GERD is primarily a clinical diagnosis, characterized by bothersome symptoms of heartburn and acid reflux. It is associated with decreased health-related quality of life and significant healthcare costs and lost productivity [13,14]. Standard treatment includes antacids, H2 receptor blockers, and proton pump inhibitors (PPIs), with the latter generally regarded as the most effective of these therapies. Nonetheless, many patients experience bothersome symptoms despite taking PPIs and there is growing concern about the overuse and potential adverse effects of PPIs [15]. Many patients who do not find relief with PPIs have functional heartburn symptoms and/or co-occurring dyspepsia symptoms (e.g., upper abdominal discomfort, bloating, and gas) that do not respond well to this class of medication [16,17].

The role of the patient-provider interaction has not been examined in the context of GERD, although placebo response rates in pharmaceutical studies vary widely (range 3–47%; [18]). A recent report estimated that 22% of U.S. adults with GERD use herbs and dietary supplements [19]. Though the percentage that use these products specifically to address GERD-related symptoms is unknown, preliminary data suggest that some dietary supplements may reduce GERD-related symptoms [20].

We took advantage of the growing interest in CIM approaches, such as dietary supplements, to study the potential effects of the patient-provider interaction and a widely available over-the-counter (OTC) CIM product on GERD symptoms. To assess feasibility and obtain preliminary estimates of effect sizes, we conducted a pilot randomized controlled trial in which subjects were randomized to receive 1) an OTC CIM product marketed for heartburn and acid reflux symptoms, Acidil, or placebo and also to 2) one of two different types of patient-provider interactions—one more typical of a conventional medical visit and another modeled after a CIM provider consultation.

## Methods

### Study design

The study was planned as a 2x2 factorial design pilot feasibility trial to investigate the effects of a widely available OTC CIM product, Acidil, and two types of patient-provider interactions, on symptoms and quality of life in patients with gastroesophageal reflux (NCT01915173). Potential subjects were screened for eligibility by telephone, sent a baseline seven day symptom diary, and scheduled for an initial appointment. At that visit, potential subjects who were deemed eligible and provided written, informed consent completed baseline measures and were randomized in a 1:1 fashion to receive either a standard or expanded medical visit. Following the visit, after the study physician left the room, subjects completed the Consultation and Relational Empathy measure [21] and the Borkovec and Nau Credibility Questionnaire [22], were randomized in a 1:1 ratio to receive Acidil or placebo, and received their study medication along with instructions for how to take it. Subjects were provided with a two week supply of study medication and a two-week daily symptom diary. Approximately two weeks following their initial visit, subjects returned, completed follow-up measures, were interviewed by the study research assistant regarding their experiences with the study, and then debriefed by the study physician regarding the details of the CIM product and the patient-provider interaction being studied. The study was approved by the Beth Israel Deaconess Medical Center (BIDMC) Committee on Clinical Investigation (the governing institutional review board) on March 1, 2013.

### Subjects

Adults age 18–80, fluent in written and spoken English, who endorsed heartburn symptoms 3 or more days per week for the past month were considered eligible. Individuals with Crohn’s disease, systemic sclerosis, known active ulcer disease, gastric cancer, untreated/active Barrett’s esophagitis, significant pain or difficulty with swallowing, heavy alcohol use (defined by > 6 drinks/week for women and > 13 drinks/week for men), concurrent pregnancy, dementia, or uncontrolled psychiatric disease were excluded. In addition, individuals who were unable to complete a paper symptom diary for at least 6 of 7 days, who had used homeopathy or had taken herbal products for GERD-related symptoms within the past 2 weeks, or had taken greater than 12 doses of NSAIDS within the prior 30 days (aspirin ≤ 325 mg daily was allowed) were excluded. Subjects with lactose intolerance were excluded as this compound was present in both the verum and placebo tablets. Individuals actively taking proton pump inhibitors (PPIs) or H2 receptor blockers were permitted to enroll as long as they were on stable doses for more than two weeks and still had breakthrough symptoms three or more days per week. Subjects were recruited from signs posted in the BIDMC primary care and gastroenterology clinics as well as by letters sent to patients seen in the primary care practice with an ICD–9 diagnosis of GERD. Twenty-four subjects were enrolled and followed between June 27, 2013 and April 23, 2014. Due to administrative issues it took several weeks to post the study on ClinicalTrials.gov, and the first subject was inadvertently enrolled during this period. Enrollment was subsequently halted until the study was officially listed. The authors confirm that all ongoing and related trials for these interventions are registered.

### Interventions

Subjects were randomized to two separate interventions: a standard vs. expanded physician visit and Acidil vs. placebo. The physician visit was single-blinded. Subjects were not informed that we were studying the patient-provider interaction during the consent process. All subjects were told, “the study physician will meet with you, much like a visit you would have with your regular doctor.” This intervention was revealed to them in a debriefing at the end of the study. All visits were performed by a single study physician (MLD). The research team did not know which intervention was being delivered until the physician opened a sealed envelope, generated by the study statistician, immediately prior to conducting the physician visit to minimize potentially biasing any interaction with study subjects prior to the visit.

A standard script of pre-determined questions was used for each visit type (S1 Scripts). The standard visit was based on a conventional primary care medical visit and asked questions about GERD history, symptoms, prior evaluation and treatments, and past medical history. The expanded visit included the same questions as the standard visit plus additional questions that inquired about modalities of their gastrointestinal (GI) symptoms (e.g., nature of the reflux taste, sensation of heartburn pain and/or abdominal fullness, time of day better/worse), details about non-GI symptoms, quality of sleep, the effect of the weather on symptoms, food cravings and aversions, menstrual flow, fears/phobias, and overall temperament. A brief and identical physical exam was included in both interventions. The study physician maintained equal empathy in both groups (e.g., kind and friendly manner, maintained eye contact, active listening and repeating back the patient’s words, expressions of empathy, and confidence that the study treatment would be effective; [4]). Thus, the main differences between the visits were the length of time spent with the subject and the additional questions that were asked. All study visits occurred in the BIDMC clinical research center.

The Acidil vs. placebo randomization was double-blinded (neither the subject nor the research team knew the allocation assignment). The randomization code was maintained by the study statistician (RBD) and the BIDMC research pharmacy. Subjects were randomized using permuted blocks randomization with randomly varying block sizes of four or eight. Acidil (purchased from Boiron, Newton Square, PA; FDA IND117358) is a combination homeopathic product available OTC. It contains four homeopathic medicines (Abies nigra, Carbo vegetabilis, Nux vomica, and Robinia pseudoacacia), each in a 4C potency (10^−8^ dilution from the original starting tincture). Each of these medications is plant-derived and has indications for heartburn and/or dyspepsia symptoms in the homeopathic materia medica. To maintain blinding, Acidil or placebo (purchased from RxHomeo, Dover, DE) tablets similar in taste and appearance were repackaged into blister packs by the BIDMC research pharmacy. Subjects were instructed to allow 2 tablets to dissolve under the tongue 3 times per day, approximately 15 minutes prior to meals. All subjects also received rescue antacid (Gelusil: aluminum hydroxide, magnesium hydroxide, simethicone) that they were permitted to take for severe breakthrough symptoms. Subjects were informed that they would be randomized to a “natural supplement containing 4 plant-based ingredients in a low dose” or a placebo. They were informed of the details of the product during the debriefing at the end of the study.

### Measures

Subjects completed a daily symptom diary for seven days prior to enrollment and during the two week study period as previously described [23,24]. They recorded the severity of nine different GERD and dyspepsia-related symptoms (daytime heartburn, nighttime heartburn, acid regurgitation, upper abdominal pain, fullness after eating, early satiety, flatulence, belching, and nausea) according to a five point scale: none, mild, moderate, severe, or very severe. The total daily GERD symptom severity was based on the sum of scores assessing severity of daytime heartburn, nighttime heartburn, and acid reflux. Scores on the last six questions were similarly used to assess the severity of co-occurring dyspepsia symptoms. The study diary also asked subjects to track how many tablets of study medication and rescue antacid they used and any other symptoms that they experienced. At the first study visit, subjects completed a demographic questionnaire, the Complementary and Alternative Medicine Beliefs Inventory [25] to assess beliefs about CIM treatment approaches, and the Consultation and Relational Empathy measure [21] after the standard or expanded visit to assess their perceptions of relational empathy during the consultation. They also completed the Gastroesophageal Reflux Disease Health Related Quality of Life instrument (GERD-HRQL,[26]) and the Gastrointestinal Symptom Rating Scale (GSRS, [27]) at both study visits. We used the Borkevec and Nau credibility questionnaire [22] immediately before and after the physician visit and again at the second study visit to assess subjects’ expectations and beliefs about the treatment they received.

### Outcomes and statistical analyses

The study was primarily designed to assess feasibility and obtain estimates to calculate sample size for future studies. With 24 subjects, we calculated a 45–74% power to detect a 30–40% difference in responders between groups in the pre-specified primary outcome measure, the percent of subjects with a 50% or greater improvement in GERD symptom severity from the daily symptom diaries comparing the 7 day baseline to the last 7 days of the study. Given the small sample size and limited power, we also pre-specified a secondary exploratory analysis using the GERD symptom severity score as a continuous variable to evaluate for potential trends. Additional secondary outcomes included changes in dyspepsia symptoms based on the daily symptom diary and changes in scores on the GERD-HRQL and GSRS reflux subscale.

Study data were collected on paper and entered twice by a research assistant into REDCap electronic data capture tools hosted at BIDMC [28]. All discrepancies between the two entries were resolved by another research team member. REDCap (Research Electronic Data Capture) is a secure, web-based application designed to support data capture for research studies, providing 1) an intuitive interface for validated data entry; 2) audit trails for tracking data manipulation and export procedures; 3) automated export procedures for seamless data downloads to common statistical packages; and 4) procedures for importing data from external sources.

Primary and secondary data analyses were performed in a blinded fashion using SAS v9.4 (SAS Institute, Cary, NC USA). All analyses were conducted as intent to treat. To assess for 50% or greater improvement in GERD or dyspepsia symptom severity, we used an exact logistic model and included a term to account for the interaction between study medication and visit type. Analysis of covariance (ANCOVA) was used to determine the effect of the study medication and visit type using baseline symptom severity as a covariate and follow-up symptom severity as the outcome. The ANCOVA models also included a term for the interaction between study medication and visit type. In all models, the interaction term was dropped if it was non-significant. The normality of the residuals for the ANCOVA models was tested using the Shapiro-Wilk test and none of the models demonstrated a significant departure from normality. Wilcoxon rank sum tests were used to compare differences in visit lengths, empathy scores on the CARE questionnaire, and treatment adherence between the standard and expanded visit groups. We analyzed adherence based upon both self-report from daily symptom diaries and from returned tablets as not every subject completed both measures. Chi square tests were used to assess blinding adequacy.

## Results

### Baseline characteristics and visit lengths

**Fig 1:**
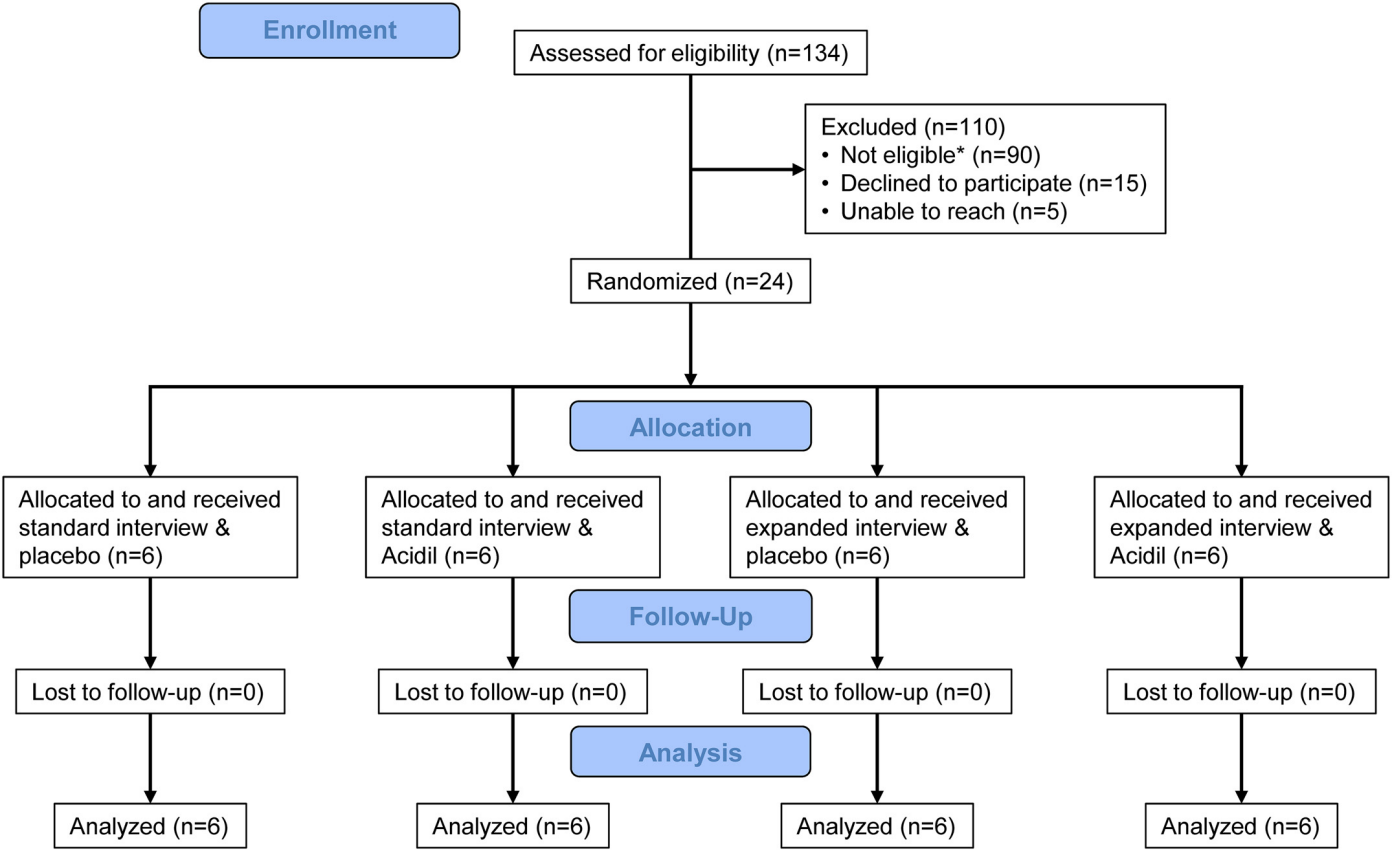
CONSORT flow diagram. *The most common reasons potential subjects were ineligible were not symptomatic enough (n = 46), and lactose intolerant (n = 19).

Twenty-four subjects consented to the study, participated in the interventions, and all 24 returned for the follow-up visit and were included in the data analysis (Fig 1). Baseline characteristics of subjects, including demographics and beliefs in complementary medicine are shown in Table 1. More subjects in the standard visit group used PPIs and more subjects in the Acidil group used antacids. Baseline and follow-up GERD symptom severity from the daily symptom diaries, dyspepsia symptom severity, GSRS reflux subscale scores, and GERD-HRQL scores are shown in Table 2. The standard visit had a median visit length of 18 minutes (range 11–32) and the expanded visit had a median visit length of 42 minutes (range 23–74; p = 0.0005).

**Table 1:** Baseline study subject characteristics (n = 24).

**Characteristic**	**Placebo Standard**	**Acidil Standard**	**Placebo Expanded**	**Acidil Expanded**	**All**
Mean Age (SD)	60 (14)	64 (6.3)	54 (9.9)	53 (12)	58 (11)
# Female	4	4	4	4	16
# Male	2	2	2	2	8
# White	4	3	3	3	13
# Black	2	2	1	2	7
# Other/Multi Race	0	1	2	1	4
Mean BMI (SD)	29 (5.5)	31 (13)	32 (7.5)	24 (3.2)	29 (8.2)
Current Smoker	1	1	1	0	3
Use PPI	3	2	1	2	8
Use H2 blocker	2	1	1	1	5
Use antacids	2	6	4	4	16
Mean CAMBI* (SD)	95 (9.2)	90 (10)	96 (15)	90 (16)	93 (13)

^#^ = number.

*CAMBI = Complementary and alternative medicine beliefs inventory [25].

**Table 2:** Mean baseline and follow-up symptom severity and health-related quality of life scores (standard deviation) and between group comparisons.

Characteristic	Placebo Standard (n = 6)	Acidil Standard (n = 6)	Placebo Expanded (n = 6)	Acidil Expanded (n = 6)	Standard vs. Expanded^~^	Placebo vs. Acidil^~^
GERD symptom severity*						
# of responders	2	0	5	4	p = 0.011	p = 0.326
Baseline	4.2 (2.1)	5.6 (2.6)	3.6 (2.2)	3.8 (2.3)		
Follow-up	2.9 (2.3)	4.2 (2.1)	0.80 (0.75)	1.7 (1.5)	p = 0.012	p = 0.195
Dyspepsia symptom severity^						
# of responders	1	1	4	4	p = 0.041	p = 1.00
Baseline	7.2 (5.1)	5.2 (3.7)	6.0 (4.6)	7.2 (2.7)		
Follow-up	5.2 (3.7)	4.3 (2.6)	1.8 (1.6)	3.3 (1.2)	p = 0.013	p = 0.663
GSRS reflux score+						
Baseline	7.8 (2.3)	7.8 (1.9)	6.5 (1.9)	6.5 (1.4)		
Follow-up	5.0 (1.7)	7.3 (1.8)	4.7 (2.0)	4.5 (1.6)	p = 0.041	p = 0.171
GERD-HRQL score#						
Baseline	24.5 (6.6)	27.0 (8.7)	26.2 (8.8)	27.5 (2.1)		
Follow-up	18.2 (4.5)	26.3 (7.8)	17.7 (3.4)	18.3 (4.9)	p = 0.076	p = 0.092

*From daily symptom diary, higher numbers signify worse symptoms, range = 0–12; # of responders = number of participants with a 50% or greater improvement in GERD symptom severity from baseline to follow-up.

^^^From daily symptom diary, higher numbers signify worse symptoms, range = 0–24; # of responders = number of participants with a 50% or greater improvement in dyspepsia symptom severity from baseline to follow-up.

^+^Gastrointestinal Symptom Related Scale, reflux subscale, higher numbers signify worse symptoms, range = 2–14.

^#^GERD-Health-Related Quality of Life instrument, higher numbers signify worse quality of life, range = 0–50.

^~^p values represent main effects from exact logistic or ANCOVA models. Trends favored the expanded and placebo interventions. All standard/expanded visit x Placebo/Acidil treatment interactions were non-significant.

Underlying data are available in Supporting Information (S1 Data).

### Treatment outcomes—GERD symptoms

In the primary outcome analysis, there was no significant difference in GERD symptom improvement from baseline to week 2 of the study between subjects receiving Acidil or placebo (p = 0.33). However, subjects who received the expanded physician visit were more likely to have a 50% or greater GERD symptom improvement compared to subjects who received the standard physician visit (p = 0.01; Table 2). In the ANCOVA analysis, subjects receiving the expanded visit were more likely to have decreased severity of GERD symptoms at follow-up (p = 0.01), and subjects receiving Acidil had a non-significant worsening of GERD symptoms at follow-up (p = 0.20; Table 2). ANCOVA analyses using the GSRS reflux subscale and the GERD-HRQL instrument showed similar trends. Individuals in the expanded visit group were also more likely to decrease the number of antacid tablets that they used for symptom management (p = 0.01).

### Treatment outcomes—dyspepsia symptoms

Many individuals with heartburn and reflux symptoms also have co-occurring dyspepsia symptoms. Using the daily symptom diary scores, we also assessed changes in dyspepsia symptoms. In logistic regression analysis, there was no significant difference in changes in dyspepsia symptoms between individuals receiving Acidil and placebo (p = 1.0), however, individuals in the expanded visit group were more likely to notice an improvement in dyspepsia symptoms compared to subjects in the standard visit group (p = 0.04). These relationships were maintained in the ANCOVA analyses when adjusting for both interventions and baseline symptom severity (p = 0.66 and 0.01, Acidil vs. placebo and expanded vs. standard visit, respectively, Table 2).

### Potential confounders

We performed several exploratory analyses to determine what factors may have contributed to the observed differences between the standard and expanded visit groups. We first analyzed scores on the CARE questionnaire. Subjects in the standard visit group gave a median score of 43 (range 35–50) for their interaction with the study physician while subjects in the expanded visit group gave a median score of 50 (range 38–50, p = 0.09). However, when we added this perceived empathy variable into the ANCOVA analysis (there was no significant co-linearity), the main effect of the visit was relatively unchanged (p = 0.03) and the CARE score was not significant (p = 0.44). Similarly, including the total length of time spent for the visit, subjects’ baseline CIM beliefs, or subjects’ post-visit confidence in the ability of the treatment to alleviate their symptoms also were not significantly associated with GERD symptom severity at follow-up (p = 0.50, 0.69, 0.80, respectively). Moreover, an ANCOVA analysis assessing whether visit type was associated with post-interview confidence in the ability of the treatment to alleviate symptoms (using baseline confidence as a covariate) demonstrated a negligible effect of the expanded visit on boosting confidence (p = 0.15).

We next analyzed the impact of race and gender by including these variables in the ANCOVA analysis. Race was non-significant (p = 0.81, white vs. non-white), however, females may have been more likely to experience an improvement in GERD symptom severity compared to males (p = 0.06).

### Additional analyses

We noted that adherence to the study supplement varied among subjects. We found that approximately 65% (based on daily symptom diary reports) to 55% (based on returned tablets) of subjects took 80% of study medication doses; 83–80% took 50% of study medication doses. As an exploratory analysis, we asked whether study medication adherence was linked to assignment to Acidil vs. placebo, and found that it was not (p = 0.96 or 0.44, self-report and tablet counts, respectively). However, adherence may have been somewhat better in the expanded visit group compared to the standard visit group (p = 0.24 or 0.05, respectively).

To assess blinding, we asked participants at the end of the study which treatment they thought they had received. The proportion of patients who thought they had received the supplement was 42% in the supplement group and 42% in the placebo group (p = 1.0), suggesting that the blinding was successful.

### Adverse events

There were no serious adverse events, or allergic reactions, during the study. We asked study subjects to record any symptoms that they experienced on their daily symptom diaries, regardless of whether they believed these to be related to the study medication. The majority of symptoms were mild and all were transient. A summary of these symptoms is reported in Table 3. A total of 35 distinct symptoms were reported. The majority of these symptoms were GI-related. There were several reports of perceived heartburn exacerbation as well as a variety of other GI symptoms (i.e., abdominal discomfort, diarrhea, constipation, flatulence, belching, nausea). Interestingly, more GI symptoms were reported in the placebo group than in the Acidil group, while there was little difference between the standard and expanded visit groups. One subject in the Acidil plus standard visit group discontinued the study medication at the beginning of the second week due to perceived side effects (nausea and worse heartburn).

**Table 3:** Subject reported symptoms by body system and group assignment.

**System**	**Total**	**Placebo**	**Acidil**	**Standard**	**Expanded**
**Gastrointestinal**	22	16	6	10	12
**Neurologic**	4	2	2	2	2
**Constitutional**	3	2	1	1	2
**Respiratory/ENT**	2	1	1	1	1
**Musculoskeletal**	2	1	1	1	1
**Cardiovascular**	1	0	1	0	1
**Psychological**	1	1	0	1	0
**Total**	35	23	12	16	19

## Discussion

In this pilot study, we found that subjects who received an expanded physician visit modeled after a visit to a CIM provider had greater improvement in both their GERD and dyspepsia symptoms compared to subjects who received a standard physician visit modeled after a conventional medical visit, irrespective of the medication they received. The significant improvements in daily symptom diary scores were corroborated by similar trends in the GSRS reflux subscale, the GERD-HRQL instrument, and reduced need for breakthrough antacids. There did not appear to be a beneficial effect of Acidil over placebo.

Visits to CIM providers are frequently longer than, and incorporate additional questions not typically encountered during, conventional medical visits. We included both of these elements in our expanded physician visit intervention. Thus, we cannot determine whether it is time, the questions themselves, or the combination that is responsible for our results. Time was not a significant covariate when added to the ANCOVA model, however, our sample size was small and we likely did not have adequate power to detect an effect. While a growing number of studies have documented the importance of the patient-provider interaction in affecting treatment outcomes [1–4], few studies specifically assess the effect of consultation length on outcomes [10]. Nonetheless, visit duration has been associated with patient satisfaction and may be associated with visit quality [29–31].

Notably, many prior studies assessing the effect of the patient-provider relationship have used a limited or low empathy intervention as the control. In contrast, we started with an empathic conventional visit and added to it and still observed an effect. We assessed the empathic quality of the patient-provider interaction in our study using the CARE measure and there was not a significant difference between groups, however, there was a ceiling effect in the expanded visit group. Moreover, CARE is specifically designed to measure empathy and does not assess other aspects of the patient-provider interaction.

Some patients state after visiting CIM providers that they felt “more heard” than in their interactions with conventional physicians [32]. Though this perception could, in part, reflect the total amount of time spent, it may also reflect a qualitative difference in the type of relationship created, a form of interpersonal healing [33], or simply the fact that having spent more time reflecting on their symptoms in a safe and non-judgmental space patients leave with a different perspective on their illness than the one they had prior to the consultation [34]. These effects may be independent of perceived empathy.

Work in the field of placebo research has suggested that beliefs, expectations, and confidence can affect how patients respond to an intervention [8]. In our study, baseline beliefs in complementary medicine did not correlate with treatment outcomes. We measured subjects’ expectations/confidence immediately before and after the physician visits. The expanded visit did not significantly affect subjects’ confidence in treatment efficacy and there was no relationship between confidence levels after the visit and treatment outcomes, though we may have been underpowered to detect a difference.

The other intervention we tested in this study was a combination homeopathic product that is available OTC. Unlike many homeopathic medicines, Acidil is not composed of “ultra-high dilutions” beyond Avogadro’s number, a source of controversy regarding the mechanism of action of homeopathic medicines. In our study, we did not detect a significant benefit to subjects who received Acidil, however, the study was not adequately powered to detect an effect. Some studies using homeopathic medicines have detected significant differences between verum and placebo on time by treatment analyses but not endpoint analyses [35], however, we did not find such a trend in our study (p = 0.29). As we used a combination product and did not individualize based on subjects’ symptoms according to standard homeopathic methodology, our study should not be construed as a test of homeopathy per se.

Limitations of our study include the small sample size and short treatment interval (two weeks), design aspects that reflect the pilot nature of the study. Another important limitation is that all visits were conducted by the same study physician. While this feature helped us to maintain consistency within and across the visit interventions, the strength of the visit effect may not be as robust with a more diverse group of clinicians. Nonetheless, all study subjects interacted with the study physician during the consent process, an element of our protocol that would be expected to bias the effect of the two visits toward the null hypothesis. Importantly, lifestyle changes that are typically advised as part of GERD symptom management (e.g., avoidance of trigger foods, eating smaller meals, eating at least 3 hours prior sleep, and elevating the head of the bed) were not explicitly discussed with subjects in any of the visits.

In summary, in this pilot randomized controlled trial of Acidil vs. placebo and two types of patient-provider interactions, we found that subjects who received a standardized expanded physician visit modeled after some types of CIM consultations experienced greater improvement in GERD symptom severity than subjects who received an empathic standard physician visit modeled after a conventional medical evaluation. There was no benefit to Acidil over placebo, however, this trial was not powered to detect a significant effect. Our results suggest that there may be a powerful salubrious effect to some complementary and integrative medicine consultations regardless of the types of interventions offered. Whether this effect is due to the amount of time spent, the types of questions asked, some aspect of the patient-provider relationship, or a combination of these effects requires further investigation. Future studies of these interactions may identify components that can be added to conventional medical visits to enhance patient outcomes.

## Supporting Information

S1 Checklist (PDF)

S1 Data (PDF)

S1 Protocol (PDF)

S1 Scripts (PDF)
