# Supplementary material for: Patient-Provider Interactions Affect Symptoms in Gastroesophageal Reflux Disease: A Pilot Randomized, Double-Blind, Placebo-Controlled Trial
Source: PLoS One. 2015 Sep 30;10(9):e0136855. doi: 10.1371/journal.pone.0136855 (PMC4589338; doi:10.1371/journal.pone.0136855)
Supplement: S1 Data — (PDF) [file pone.0136855.s002.pdf]

S1 Data - supporting information for data presented in Table 2

Subject	GSS-pre	GSS-post	DSS-pre	DSS-post	GSRSR-pre	GSRSR-post	GERD-HRQL-pre	GERD-HRQL-post
1	7.43	0.86	11.76	3.43	8	2	35	12
2	7.86	1.62	4.86	2.57	8	4	24	16
3	2.86	3.43	5.60	7.67	7	5	27	23
4	6.71	3.17	15.00	11.71	6	6	18	23
5	2.57	0.93	4.71	3.60	5	3	26	20
6	2.86	1.29	1.43	2.43	7	5	17	15
7	4.50	4.00	15.00	2.14	12	3	23	27
8	2.95	2.83	2.43	0.83	5	8	25	23
9	6.71	3.71	3.43	3.71	8	7	30	20
10	1.55	1.67	5.43	3.71	3	5	13	18
11	7.00	4.29	4.67	0.62	8	8	33	31
12	1.12	0.43	4.86	0.00	6	4	38	39
13	3.57	1.71	4.29	5.00	7	5	44	12
14	4.14	2.43	2.71	2.50	8	10	21	20
15	5.00	7.14	10.29	4.00	7	7	21	15
16	2.14	0.14	0.57	1.57	7	4	23	19
17	2.37	0.43	4.57	3.67	8	3	24	20
18	4.09	0.00	8.74	8.94	7	8	24	20
19	3.12	2.00	7.43	3.67	8	7	29	22
20	7.86	4.17	4.29	1.71	11	6	30	22
21	8.57	8.29	10.86	5.29	8	7	26	24
22	2.14	2.00	9.83	2.43	5	4	26	13
23	2.14	1.14	4.60	3.00	5	4	29	13
24	3.71	0.14	6.29	3.57	8	4	25	16

GSS =average daily GERD symptom severity
DSS = average daily Dyspepsia symptom severity
GSRSR = Gastrointestinal Symptom Related Scale, reflux subscale
GERD-HRQL = GERD Health-Related Quality of Life
Pre = baseline measure
Post = follow-up measure

Additional data available by writing to the corresponding author and submitting appropriate forms to the IRB.
