# Supplementary material for: Patient-Provider Interactions Affect Symptoms in Gastroesophageal Reflux Disease: A Pilot Randomized, Double-Blind, Placebo-Controlled Trial
Source: PLoS One. 2015 Sep 30;10(9):e0136855. doi: 10.1371/journal.pone.0136855 (PMC4589338; doi:10.1371/journal.pone.0136855)
Supplement: S1 Protocol — (PDF) [file pone.0136855.s003.pdf]

PART B
STUDY DESCRIPTION

TITLE OF PROTOCOL	Response to Supplement and Placebo in GERD		
Principal Investigator	Gloria Yeh, MD, MPH		
Co-Investigators	Michelle Dossett MD PhD, Ted Kaptchuk, Anthony Lembo MD, Roger Davis ScD		
Mailing Address	330 Brookline Ave, CO-206, Boston, MA 02215		
E-Mail Address	gyeh@bidmc.harvard.edu		
P.I.'s Telephone	617-754-1419	P.I.'s Pager	Fax:617-754-1440
Sponsor/Funding Source	None		

B1. PURPOSE OF PROTOCOL

The purpose of this study is to investigate the effects of a) a homeopathic supplement marketed for heartburn symptoms (Acidil) and b) components of the placebo response (the doctor-patient interaction and belief/expectation) on symptoms and health-related quality of life (HRQL) in patients with gastroesophageal reflux disease (GERD). This study is designed as a pilot trial to assess safety and feasibility and to provide preliminary estimates of effect sizes in anticipation of conducting a larger trial in the future.

Aim 1: To determine the safety and feasibility of conducting this 2x2 factorial design randomized controlled trial in this patient population.
Hypothesis 1: This study design will be both safe and feasible and subject recruitment will not be difficult (i.e., no significant adverse events and greater than 90% of those who enroll will complete the study protocol).

Aim 2: To determine whether changes in GERD-specific, dyspeptic, and overall symptoms and HRQL are significantly different between subjects receiving Acidil and subjects receiving an identical placebo.
Hypothesis 2a: Compared to subjects receiving placebo, more subjects receiving Acidil will experience a 50% or greater improvement in GERD symptom severity during the last 7 days of the study compared to baseline.
Hypothesis 2b: The dynamic trajectory of symptom changes over time will differ between the Acidil and placebo groups as expected according to homeopathic theory.
Hypothesis 2c: Subjects receiving Acidil will report greater improvement in disease-specific and overall quality of life compared to the placebo group.

Aim 3: To determine whether changes in GERD-specific, dyspeptic, and overall symptoms and HRQL are significantly different between subjects receiving an expanded clinical interview similar to a homeopathic consultation (enhanced physician-patient interaction) vs. a standard allopathic interview, and to determine whether any treatment responses are mediated by preconceived beliefs and expectations regarding treatment efficacy.
Hypothesis 3a: Compared to subjects receiving the standard clinical interview, more subjects receiving an expanded clinical interview will have a 50% or greater improvement in GERD symptom severity during the last 7 days of the study compared to baseline.

Hypothesis 3b: Subjects receiving an expanded clinical interview will report greater improvement in disease-specific and overall quality of life compared to subjects receiving a standard clinical interview.
Hypothesis 3c: Subject beliefs and expectations pre-treatment will partially explain changes in GERD and dyspeptic symptoms in the expanded vs. standard interview groups but not in the Acidil vs. placebo groups.

B2. SIGNIFICANCE AND BACKGROUND FOR THE STUDY

Gastroesophageal Reflux Disease (GERD)

Twenty to thirty percent of Americans experience GERD-related symptoms consisting of heartburn and/or acid reflux at least weekly (1). Although proton pump inhibitors (PPIs) are the third largest class of pharmaceutical drug sales in the United States (2), there is increasing concern about the overuse and negative side effects of these medications. A growing body of evidence has linked PPI use with increased risk of C. difficile infections, small bowel bacterial overgrowth, osteoporosis, nutritional deficiencies, and potential drug-drug interactions related to CYP450 enzyme metabolism (2, 3). Moreover, many patients continue to experience distressing GI symptoms despite taking PPIs. A number of these patients have functional heartburn symptoms and/or co-occurring dyspeptic symptoms (e.g., upper abdominal discomfort, bloating, and gas) that do not respond well to this class of medication (4).

Placebo Response

A review of placebo-controlled PPI trials suggests that the magnitude of the placebo response in patients with GERD averages around 19% (range 3-47%, (5)). However, the factors that influence the magnitude of this response in this patient population are poorly understood. Data from studies of other conditions suggests that the placebo response is a complex phenomenon that includes: natural history & regression to the mean, expectancy & belief (involving both the patient and the doctor), classical conditioning, learning, and reward pathways in the brain, and the quality of the therapeutic encounter & ritual (6, 7). Recent work by Ted Katpchuk, Tony Lembo, and colleagues has demonstrated that in patients with irritable bowel syndrome, the magnitude of the placebo effect can be augmented by enhancing the quality of the therapeutic interaction (7) and that a genetic polymorphism in the catechol-o-methyltransferase gene correlates with this response (8). To date, there have been no studies examining the components of the placebo response in patients with GERD.

Complementary Medicine

Many patients are interested in exploring alternative treatments for their medical conditions aside from traditional pharmaceutical drugs. In 2007, 38% of US adults had used some form of complementary medicine and spent $33.9 billion out-of-pocket on practitioner visits and over the counter (OTC) products (9). The majority of these OTC products have not been formally tested for safety or efficacy. The same survey also revealed that homeopathy is one of the top 10 complementary modalities used in the US, with the population spending nearly $2.9 billion out of pocket on homeopathic medicines and services.

Homeopathy

Homeopathy is a system of medicine that was founded in 1796 by the German physician Samuel Hahnemann. It has grown to become one of the most popular medical systems used across the world (10). Indeed, the Swiss government recently reviewed the evidence for homeopathy and concluded that

there was sufficient data regarding safety, efficacy, and cost-effectiveness to warrant coverage of homeopathic services by the national health system (11).

Nonetheless, some authors believe that the effects of homeopathy are due to enhanced placebo effects and an augmented doctor-patient interaction. In classical homeopathy, a typical homeopathic consultation can last 1-2 hours and involves the exploration of domains of illness not usually discussed in a typical allopathic medical consultation. These extensive consultations are deemed necessary to gather adequate information to properly individualize a patient's treatment, a process that is complex and labor intensive.

To make homeopathy more accessible to the lay public, some homeopathic pharmaceutical companies have created formulations for common conditions that combine multiple homeopathic medicines commonly used to treat a set of symptoms into a single product, or supplement, marketed to treat a specific indication (e.g., heartburn, insomnia, or seasonal allergies). While combination homeopathic products do not conform to the standards of classical homeopathy (in which only a single homeopathic medicine is prescribed at a time and typically higher dilutions are used), they are widely sold over the counter in the U.S. and used by the public to treat a variety of conditions. The efficacy of many of these products has not been verified in the peer-reviewed literature.

To date, there are no trials of homeopathy specifically for the treatment of GERD, however, homeopathic providers often prescribe for GERD-related symptoms (personal communication). There are trials suggesting possible benefit of individualized homeopathy for dyspeptic symptoms (12) as well as functional medical conditions such as fibromyalgia (13).

Homeopathic medicines are generally regarded as very safe, due to the low concentrations of active ingredients present, with adverse event rates generally estimated to be 3% or less and serious adverse events extremely rare (11, 14). In the United States, homeopathic medicines are regulated by the FDA under the 1938 Food, Drugs, and Cosmetics Act (15). The majority of homeopathic medicines are available OTC without a prescription required. According to the manufacturer (Boiron) of the product to be used in this study (Acidil), there have been no adverse event reports for this supplement over the last 10 years in the United States.

Despite the relative safety of homeopathic medicines, certain types of treatment reactions are commonly recognized by the homeopathic community. These include aggravations and provings. An aggravation is a transient aggravation of a patient's symptoms followed by a marked improvement in symptoms and is felt to be a positive prognostic sign of healing. A proving occurs when a patient manifests symptoms that the homeopathic medicine is designed to treat and usually occurs when a patient has received too much of the medicine and/or the medicine is poorly matched to the patient's symptoms. Aggravations and provings may explain the dynamic oscillations seen in patients' symptoms in the homeopathy arms, but not the placebo arms, of some randomized controlled trials (16, 17). These observations complicate the interpretation of treatment outcomes and may account for some inconsistent results in prior homeopathic studies. Thus, in our study, we propose to monitor dynamic changes in symptoms over time as well as conduct endpoint analyses.

B3. DESCRIPTION OF RESEARCH PROTOCOL

A. Study Design – Overview, Methods, Procedures

Overview

	Acidil	Placebo
Standard Interview	Group 1	Group 2
Expanded Interview	Group 3	Group 4

We propose to conduct a single-center, pilot feasibility, 2x2 factorial design randomized controlled trial assessing the effect of a homeopathic supplement (Acidil) and components of the placebo response (doctor-patient interaction and beliefs/expectations) on symptoms and HRQL in patients with GERD over a 2 week time interval. The Acidil vs. placebo intervention will be double-blinded and the standard vs. expanded interview intervention (doctor-patient interaction) will be single-blinded. Randomization will be by permuted block randomization with block sizes of 4 and 8. We plan to enroll 6 patients into each group for a total of 24 subjects in the study according to the adjacent scheme. Subjects will be evaluated in the GCRC at BIDMC at two timepoints: baseline and at 2 week follow-up.

Detailed Study Procedures, Timeline, Testing, and Outcome Measures

An overview of the study timeline is shown below.

Figure 1: Study Timeline

Time 2: +2 weeks, 2nd study visit
Collect symptom diary
Blood draw
Questionnaires
Exit interview

Time 0: -1.5-3 weeks
Subject calls
Telephone screen for eligibility
Instructions for symptom diary

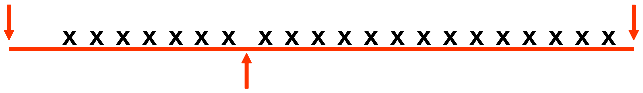

Time 1: 1st study visit
Final eligibility assessment, formal consent
Blood draw
Questionnaires (symptoms, QOL, attitudes to CAM)
Randomization
Standard or expanded interview
Questionnaires (credibility, empathy)
Study drug dispensed

x = daily symptom severity and frequency and medication use diary

Telephone Screening (Time 0: -1.5 to -3 weeks):

When a potential study subject calls to inquire about participating in the study, they will be provided with further information over the phone (Appendix A) about the study and their questions answered. If they are interested in participating, potential subjects will undergo a brief telephone screen to assess eligibility (Appendix A). If they are a BIDMC patient, their OMR record will be reviewed to confirm eligibility. If they pass the telephone screen, they will be informed that they are likely eligible for the study, but will need to complete a baseline symptom diary and have a final in-person eligibility assessment at BIDMC. If they wish to proceed, instructions for completing the baseline symptom diary

will be explained, a diary will be mailed to the potential subject, and appointments scheduled for a baseline evaluation and 2 week follow-up. Potential subjects will be called several days after the diary is mailed to ensure that they received it and that the instructions for completing the diary are clear.

First Study Visit (Time 1):

When a potential subject arrives at the study site, the diary will be reviewed to ensure that it meets eligibility criteria (symptoms at least 3 days out of 7 and symptom frequency and severity recorded on at least 6 days of 7) and the individual will undergo a final in person eligibility assessment reviewing the criteria discussed during the phone screen. Subjects deemed ineligible will be thanked for their effort, informed that they are not eligible, receive parking validation and be dismissed.

Eligible subjects will be formally consented, have blood drawn (if they opt to participate in the repository study described in a separate application), and complete a series of baseline questionnaires (demographics, medication and supplement use, GSRS, Nepean Dyspepsia Index, GERD-HRQL, SF-12, MYMOP, credibility questionnaire, CAMBI, see Table 1 below). Next, subjects will be randomized to one of the 4 treatment groups and receive either the standard or expanded interview in a single-blinded fashion (the study physician will be aware of assigned interview type, but the subject will not). The clinical interview will be audio and video recorded (pending subject permission) to permit subsequent assessment of fidelity to the treatment protocol.

After the interview, subjects will complete additional questionnaires (CARE, credibility questionnaire, see Table 1). Finally, subjects will receive the study medication (Acidil or placebo) in a double-blinded fashion (neither the study physician nor the subject will be aware of treatment assignment) and rescue antacid. The study physician, or another member of our research team, will provide medication education. All subjects will receive the same instructions for how to take the study medication and rescue antacid, will be provided with a new study diary, and their 2 week follow-up appointment will be confirmed. Study subjects will be contacted 7 days following the first study visit to confirm that they are taking the study medication, completing their daily diary, and not experiencing any marked adverse effects. The first study visit is anticipated to last 1-2.5 hours.

Intervention and Control Groups:

Standard and Expanded Intake Groups

Subjects assigned to groups 1 and 2 will receive a standard allopathic clinical interview (Appendix B) by the study physician inquiring into the nature and history of their GERD and dyspeptic (if present) symptoms and prior and current treatments undertaken to control symptoms, including any complementary treatments. The study physician will also review the subject's symptom diary and the study questionnaires completed immediately prior to the interview and conduct a brief physical exam. The interview is expected to last 20-30 minutes.

Subjects assigned to groups 3 and 4 will receive an expanded clinical interview (Appendix B) by the study physician similar to the type of interview performed by classical homeopathic physicians. In addition to the above evaluation, the expanded interview will also include questions inquiring about any additional symptoms or diagnoses that the subject currently has, the subject's temperament, likes and dislikes, effects of weather on symptoms, and sleep. The expanded interview is expected to last approximately 1 hour, though for medically complex patients it could last as long as 1.5 hours.

The same study physician (Dr. Dossett) will be conducting all study interviews and thus will not be blinded to this aspect of study group assignment, but will remain blinded to study medication assignment. Dr. Dossett has been receiving training in classical homeopathic case taking and analysis and will thus be able to mimic the types and depth of questions typical for a homeopathic consultation. As this aspect of the intervention is primarily about the length of time spent with the subject, and depth of questions asked, there will be no overt attempt to vary the amount of empathy between the two

interview types. However, we will be measuring perceived empathy using the CARE questionnaire. Engagement with a warm, caring provider is known to improve response to treatment (Kaptchuk). Whether consultation length affects treatment response and whether this effect is mediated through perceived empathy is unknown. Both the standard and expanded intakes will be audio and video recorded (physical exam component audio only so that the study camera does not need to be moved and to preserve subject modesty) so that they can be analyzed later by an independent evaluator to ensure fidelity to the study protocol. Consent for recording will be separate from the study consent so that a subject may still consent to study participation even if he/she declines audio and/or video recording.

At the time of consent, study subjects will not be informed about the two different interview types or that part of the study involves testing the effect of the doctor-patient interaction on response to treatment. Concealment of these aspects of the study protocol is essential to maintaining the validity of the study as data suggests that patients responses change when they know that the doctor-patient interaction is being studied (6, 18). Subjects will be informed in the consent document that their interview with the study physician could last anywhere from 20-90 minutes. We plan to notify subjects at the end of the study, after the exit interview, that there were two possible types of physician interviews, that this aspect of the study was concealed from them, and of the rationale for why we did so. A script outlining this planned debriefing is present in Appendix F. We believe that this aspect of the intervention is minimal risk and that our plan meets the federal criteria for permitting alteration of some elements of informed consent (19).

Acidil and Placebo Groups

Subjects assigned to groups 1 and 3 will receive a low potency combination supplement known as Acidil (20), commonly sold over the counter at health food stores. Acidil contains four homeopathic medicines (Abies nigra, Carbo vegetabilis, Nux vomica, and Robinia pseudoacacia), each in a 4C potency (10^{-8} dilution from the original starting tincture). Each of these medications is plant derived, has indications for heartburn and/or dyspeptic symptoms in homeopathic materia medica, and is listed in the Homoeopathic Pharmacopoeia of the United States, the official compendium of homeopathic drugs that are licensed for sale in the United States (21, 22). In addition, we will be filing an IND with the FDA to use this supplement. While there are no previously published studies of Acidil in the peer-reviewed literature, our rationale for studying Acidil is that it is a popular over the counter product widely sold in the US, France (as Gastrocynesine), and several other European countries for heartburn and related symptoms. This study thus begins to address a practical question regarding a commonly used and available supplement.

Subjects assigned to groups 2 and 4 will receive placebo pills similar in taste, appearance, and packaging to Acidil. All subjects will receive the study medication in a double-blind fashion. All subjects will also receive rescue antacid (Gelusil: aluminum hydroxide, magnesium hydroxide, simethicone) to take for breakthrough symptoms.

In addition to receiving oral instructions for how to take the study medication, subjects will also be provided with written instructions for how to take the study medication and rescue antacid (Appendix C). In general, they will be advised to allow 2 tablets to dissolve under the tongue 3 times per day, 15 minutes prior to meals. In the event of the situations below, they will be advised the following:

- If they experience severe GERD-related symptoms despite taking the study medication, they should take the rescue antacid, Gelusil, for symptom relief and continue taking the study supplement as prescribed.
- If they experience an immediate and marked worsening of their symptoms, they should follow the above instructions. If their symptoms do not improve after 2 additional doses, they should stop taking the supplement and call the study center.

- If they experience an improvement in symptoms, they should continue taking the study supplement as prescribed.
- If they experience an initial improvement in symptoms and subsequent symptom worsening, they should decrease dosing frequency to once or twice daily. If symptoms further worsen, they should stop the supplement entirely and call the study center.
- Subjects will be counseled to call the study center if they have any questions about their symptoms or dosing of the supplement.

During the consent process, we will not inform study subjects of the name of the study supplement or that it is a homeopathic product. Acidil is widely available over the counter, inexpensive (less than $10 for 60 tablets), and we are unable to obtain a placebo that is an exact match for the product. As such, it would be very easy for subjects to unblind themselves during the trial and invalidate the results of the study. Furthermore, it is likely that most of the lay public do not know exactly what homeopathy is, and if they were to read the word "homeopathy" or "Acidil" in the consent document, they would likely search the internet or seek out additional information after the first study visit. Beliefs and expectations are known to influence treatment responses, and we are interested in studying how the length of time and depth of questions asked during a physician interview affects patients' beliefs about the effectiveness of a treatment. Given the controversy surrounding homeopathy and the variable quality of information on the internet, subjects' beliefs about the treatment would likely change and could weaken the difference between the effect of the standard vs. expanded interviews. Finally, many people would argue that calling Acidil "homeopathy" is also deceptive as classical homeopathy relies on the prescription of a single homeopathic medicine at higher dilutions closer to, or beyond, Avogadro's number. The product being studied contains a combination of homeopathic medicines at relatively low diluations (i.e., more concentrated than typical homeopathic medicines), and thus is more like a supplement than traditional homeopathy. We believe that the way we have described this product in the consent (a combination of 4 plant-based products, each in a low dose) is the best way to describe the intervention while maintaining the validity of the study. After the exit interview subjects will be informed of the name of the study supplement (Appendix F). However, because of the need to maintain the study physician's blinding, they will not be informed whether they received Acidil or placebo until after the trial is over via a letter (Appendix F).

Second Study Visit (Time 2 - approximately 2 weeks after first visit):

Study diaries will be collected and subjects will provide a second blood specimen (if they consent to the repository study) and complete study questionnaires (medication and supplement use, GSRS, Nepean Dyspepsia Index, GERD-HRQL, SF-12, MYMOP, treatment satisfaction, credibility questionnaire, predicted treatment assignment, belief in homeopathy, see Table 1). Subjects will then have a brief semi-structured exit interview with the study physician or another member of the research team (Appendix D) inquiring about their use of the study medication, symptoms, and experience in the trial. After the interview, subjects will be debriefed regarding the concealment of the doctor-patient interaction study in this trial and will be informed of the name of the study supplement (Appendix F). The second study visit is anticipated to last approximately 1 hour. Subjects who continue to experience symptoms at the end of the trial will be referred to their PCP for further evaluation.

Testing and Outcome Measures:

All testing will be conducted at the BIDMC GCRC on east campus. A schedule of testing is outlined in the table below. All testing will be conducted by study staff and physicians who are blinded to treatment assignment (except for the study physician who will know whether the patient is receiving the standard or expanded interview).

Study Measures and Timepoints						
#	Measure/Procedure	Description/Rationale	0	1a	1b	2
1	Study Diary	Daily dairy assessing GERD, dyspeptic, & other symptoms and medication use	Throughout			
2	Anthropomorphic data	Height, weight, BMI		X		
3	Demographic Questionnaire	E.g., age, gender, race/ethnicity, smoking status, alcohol & caffeine use		X		
4	Medication Questionnaire	List of current medications & supplements		X		X
5	GSRS - Gastrointestinal Symptom Rating Scale (23)	15 item measure of GI symptoms (5 subscales), validated in patients with GERD		X		X
6	GERD-HRQL (24)	11 item measure of GERD HRQL		X		X
7	Nepean Dyspepsia Index (25)	10 item measure of dyspepsia-specific HRQL		X		X
8	SF-12 (26)	12 item HRQL instrument in 6 domains		X		X
9	MYMOP – Measure Yourself Medical Outcome Profile (27)	7 item measure of impact of treatment on main symptoms and overall well-being		X		X
10	Treatment satisfaction (28)	4 item measure for satisfaction with GERD treatment				X
11	Borkovec & Nau Credibility Questionnaire (29)	4 item measure of confidence in treatment efficacy		X	X	X
12	CARE – Consultation and Relational Empathy (30)	10 item measure of patient's perception of empathy from the physician			X	
13	CAMBI - Complementary and Alternative Medicine Beliefs Inventory (31)	17 item measure of beliefs about complementary and holistic health		X		
14	Treatment assignment	2 questions about predicted treatment assignment and certainty				X
15	Belief in homeopathy	2 questions about whether patient has heard about homeopathy and what they think of it				X
16	Interview	Semi-structured exit interview to assess experience with the trial				X
0 = Telephone screening<br>1a = 1st study visit, before the standard/expanded interview<br>1b = 1st study visit, after the standard/expanded interview<br>2 = 2nd study visit, 2 weeks later, end of study						

Copies of all instruments are in Appendix D.

Primary Measures

Study diary: GERD and dyspepsia symptom frequency and severity will be assessed by reviewing daily

study diaries similar to those used in prior trials of pharmaceuticals for GERD (28, 32-34). The study diary includes a 5 point scale assessing the severity of symptoms (daytime heartburn, nighttime heartburn, acid regurgitation, epigastric pain, fullness after eating, early satiety, flatulence, belching, and nausea). In addition, the study diary will assess the presence of any other non-GERD, non-dyspeptic symptoms and their severity, potential side effects, and the amount of study medication and rescue antacid used.

Secondary Measures
GERD-HRQL: The GERD-HRQL questionnaire is a validated 11 item scale for assessing the impact of GERD symptoms on HRQL (24).
Nepean Dyspepsia Index: The Nepean Dyspepsia Index is a validated 10 item scale for measuring dyspepsia-specific HRQL in 5 domains (25).
SF-12: The SF-12 is a validated 12 item questionnaire for measuring overall HRQL in 6 domains (26).
MYMOP: The Measure Yourself Medical Outcome Profile (MYMOP) is a validated 7 item measure of the impact of treatment on symptom severity and overall well-being (27). It has been used in a variety of studies of CAM and conventional modalities.
Treatment satisfaction: A 4 question assessment of satisfaction with GERD treatment (28).

Other Measures
Study diary: Changes in non-GERD, non-dyspeptic symptoms will be assessed by reviewing the study diaries and the MYMOP questionnaire.
Study diary: Study drug and rescue medication use will be assessed by reviewing the study diaries.
Medications and supplement questionnaire: All subjects will complete a medication and supplement use questionnaire at their first and second study visits to determine if there have been any medication or supplement changes during the study period. The questionnaire will be reviewed by the study physician with the subject.
GSRS: The Gastrointestinal Symptom Rating Scale is a validated 15 item instrument for measuring the severity of gastrointestinal symptoms (23). It contains 5 subscales: abdominal pain, reflux syndrome, diarrhea syndrome, indigestion syndrome, and constipation syndrome.
Credibility questionnaire: To assess how subjects' views regarding the study treatment change over time, we will have subjects complete the Borkovec and Nau credibility questionnaire (29) before and after the clinical interview and at the 2 week follow-up visit.
CARE: To assess subjects' perceptions of their intake with the study physicians, they will complete the Consultation And Relational Empathy questionnaire (30), a validated 10 item measure for assessing the quality of the patient encounter and perceptions of empathy from the treatment provider.
CAMBI: To assess subjects' beliefs and expectations regarding complementary medicine, we will have them complete the Complementary and Alternative Medicine Beliefs Inventory, a validated 17 item questionnaire (31).
Treatment assignment: At the second study visit, we will ask participants whether they think they were assigned to the Acidil or placebo groups and how certain they are of this belief.
Belief in homeopathy: We will ask subjects 2 questions about their use of, and belief in, homeopathy during the second visit to better understand perceptions regarding this treatment modality in this population.
Interview: We will conduct a semi-structured interview to assess subjects' experiences with the trial for qualitative analysis.

<u>Phone calls:</u>
When subjects call indicating an interest in participating in the study, we will attempt to call them

back up to 5 times. If the potential subject does not answer or return calls, no further attempts to contact that individual will be made.

If a subject is unable to attend, or misses, the second clinic visit, we will attempt to contact the subject up to 5 times to reschedule and/or complete the follow-up study questionnaires by phone and/or mail.

Monitoring of Adverse Events and Patient Safety:

Any and all adverse events will be reported to our independent safety and monitoring board. No interim analyses of study efficacy are planned due to the pilot nature of this study.

B. Statistical Considerations

Sample Size Justification:

We will screen a sufficient number of subjects to consent/enroll 24 subjects total into the study, 6 subjects in each of the 4 groups. This number was determined to ensure that we can complete enrollment within a 6-12 month time frame to assess study feasibility as our primary outcome.

As this is a pilot feasibility study, we do not anticipate having adequate power to show significant differences in treatment effects between groups, but do hope to generate estimates of effect sizes and demonstrate trends. Our chief secondary outcome will be the percent of subjects with 50% or greater improvement in heartburn symptom severity for the last 7 days of the study compared to baseline (compare groups 1+3 vs. groups 2+4 [Aim 2a] and groups 1+2 vs. groups 3+4 [Aim 3a]). We will also assess the percent of subjects with dynamic changes in heartburn symptom severity during the study period compared to baseline (compare groups 1+3 vs. groups 2+4 [Aim 2b]).

Table 2 illustrates power estimates using the Fisher exact test for between group differences ranging from 0.25 – 0.4 for an alpha of 0.05 and sample sizes of 24 subjects and 20 subjects per group (combining groups 1+3 and 2+4 or groups 1+2 and 3+4 for all analyses). We anticipate that we will have 30-50% power to detect a difference between groups. These power calculations are based on the assumption of no interaction effect.

Table 2: Power Estimates.

p1*	p2*	Difference	Power, n=24	Power n=20
.25	.5	.25	.325	.261
.35	.6	.25	.307	.247
.45	.7	.25	.313	.252
.2	.5	.3	.479	.391
.3	.6	.3	.445	.362
.25	.6	.35	.595	.497
.35	.7	.35	.585	.488
.2	.6	.4	.740	.640
.3	.7	.4	.719	.619

*p1 and p2 are the proportion of subjects in comparison 1 vs. comparison 2.

Data Analysis:

All study data will be transcribed from paper surveys and entered into a REDCap database for data management. We will perform random screening of entered data to ensure accuracy. At the time that the REDCap database is translated into a SAS database for data analysis, the 2 main intervention codes (i.e., Acidil vs. placebo and standard vs. expanded interview) will be will be recoded by a member of the study team who did not have any subject contact (Dr. Davis) so that the principle data analyzer (Dr. Dossett) is blinded to all study group assignments during the data analysis process. Initial analysis will include descriptive statistics assessing the baseline demographics, GERD and dyspepsia symptom frequency and severity, and HRQL among subjects in each of the 4 groups. All analyses described below will be intent-to-treat analyses and include an interaction term to account for possible interaction of the 2 main interventions in this study. The SAS database will not include any PHI and will be stored on the study investigator's H drive and thus will be password protected and behind a secure firewall.

Hypothesis 1: This study design will be both safe and feasible and subject recruitment will not be difficult.

Study safety will be established if there are no major adverse events attributed to Acidil and no statistically significant increase in minor adverse events between the Acidil and placebo groups. Feasibility will be established if we can demonstrate recruitment and retention of >90% of subjects within the pre-specified 6-12 month time frame.

Hypothesis 2a: Compared to subjects receiving placebo, more subjects receiving Acidil will experience a 50% or greater improvement in GERD symptom severity during the last 7 days of the study compared to baseline.

A common measure for assessing treatment efficacy in GERD trials is assessment of the percent of subjects with a 50% or greater improvement in heartburn symptom severity (based on summation of symptom scores over time) using daily symptom diaries. We will perform this assessment by combining responses to the daytime heartburn, nighttime heartburn, and acid regurgitation questions on the daily symptom dairies for the last 7 days of the study compared to baseline. We will compare groups 1 and 3 with groups 2 and 4 using a logistic regression analysis and including an interaction term for the two study interventions. If there is no difference between groups, we will perform a secondary analysis using the GERD symptom severity score as a continuous variable to evaluate for potential trends.

Hypothesis 2b: The dynamic trajectory of symptom changes over time will differ between the Acidil and placebo groups. These differences may manifest as oscillations in symptoms in the Acidil group during the 2 week trial period, as well as changes in non-GERD and non-dyspeptic symptoms.

To assess for dynamic changes in treatment effects over time that may not be visible in end-point analyses such the one described in 2a above, we will perform a repeated measures analysis using generalized estimating equations and incorporating a time by treatment interaction term that will allow us to examine changes in GERD and dyspepsia symptom severity for the Acidil (groups 1+3) vs. placebo (group 2+4) group over the entire 2 week study period as well as look for differences in the number and frequency of non-GERD and non-dyspeptic symptoms based on data in the daily symptom diaries.

We will also assess the mean number of days without heartburn symptoms during the trial period for groups 1+3 and 2+4 as well as the mean number of days without dyspeptic symptoms during the

trial period (again groups 1+3 and 2+4) using the data from the GERD symptom dairy.

Hypothesis 2c: Subjects receiving Acidil will report greater improvement in disease-specific and overall quality of life compared to the placebo group.

We will compare GERD-HRQL, Nepean Dyspepsia Index, and SF-12 scores between the Acidil and placebo groups (1+3 and 2+4), incorporating an interaction term for the two main treatment effects, using ANOVA.

Hypothesis 3a: Compared to subjects receiving the standard clinical interview, more subjects receiving an expanded clinical interview will have a 50% or greater improvement in GERD symptom severity during the last 7 days of the study compared to baseline.

We will perform an analysis analogous to that for hypothesis 2a above by combining responses to the daytime heartburn, nighttime heartburn, and acid regurgitation questions for the last 7 days of the study compared to baseline on the daily symptom dairies comparing groups 1 + 2 with groups 3 + 4 using a logistic regression analysis and including an interaction term for the two study interventions.

Hypothesis 3b: Subjects receiving an expanded clinical interview will report greater improvement in disease-specific and overall quality of life compared to subjects receiving a standard clinical interview.

We will compare GERD-HRQL, Nepean Dyspepsia Index, and SF-12 scores between the standard (groups 1 + 2) and expanded (groups 3 + 4) interview groups, incorporating an interaction term for the two main treatment effects, using ANOVA.

Hypothesis 3c: Subject beliefs and expectations pre-treatment will partially explain changes in GERD and dyspeptic symptoms in the expanded vs. standard interview groups but not in the Acidil vs. placebo groups.

We will perform a logistic regression analysis including subjects' pre-treatment belief in CAM and scores on the credibility questionnaire using greater than 50% improvement in heartburn symptom frequency or severity during the last 7 days of the study compared to baseline (based on the daily symptom diaries) as the outcome. Due to the small sample size, we will be unable to adjust for potential confounders.

Additional Exploratory Analyses:

1. Difference between the Acidil and placebo groups (1+3 and 2+4) and the standard vs. expanded interview groups (1+2 vs. 3+4) in the mean number of rescue antacid tablets required for symptom relief.
2. Difference between the Acidil and placebo groups (1+3 and 2+4) and the standard vs. expanded interview groups (1+2 vs. 3+4) in changes in dyspeptic symptoms during the last 7 days of the trial compared to baseline.
3. Difference between the Acidil and placebo groups (1+3 and 2+4) and the standard vs. expanded interview groups (1+2 vs. 3+4) in GSRS scores between the first and second study visits.
4. Difference between the Acidil and placebo groups (1+3 and 2+4) and the standard vs. expanded interview groups (1+2 vs. 3+4) in changes in non-GERD and non-dyspeptic symptoms during the last 7 days of the trial compared to baseline.
5. Changes in scores for symptom severity and well-being on the MYMOP questionnaire pre-post for the Acidil vs. placebo groups (1+3 and 2+4) and the standard vs. expanded interview groups (1+2 vs. 3+4).
6. Differences in GERD treatment satisfaction scores for the Acidil vs. placebo groups (1+3 and 2+4) and the standard vs. expanded interview groups (1+2 vs. 3+4).

7. Differences in scores on the CARE measure for the standard vs. expanded interview groups (1+2 vs. 3+4).
8. Changes in scores on the credibility questionnaire over time by treatment assignment.
9. Changes in medications during trial period for each group.
10. Percent of subjects in groups 3 and 4 whose symptoms match those for one of the homeopathic medicines in the combination homeopathic supplement based on either a clinical or classical homeopathic case analysis approach.

C. Subject Selection

Eligibility is defined by the following:

Inclusion criteria:

1. Adult humans age 18-80.
2. Fluency in written and spoken English.
3. Heartburn symptoms 3 or more days per week for the past month.

Exclusion criteria:

*1. Subjects who have taken a proton pump inhibitor (PPI) or H2 receptor blocker within 2 weeks of the initial study visit.

2. Subjects with Crohns disease, systemic sclerosis, known active ulcer disease, gastric cancer, Barrett's esophagitis, significant pain or difficulty with swallowing, heavy alcohol use (defined by > 6 drinks/week for women and > 13 drinks/week for men), concurrent pregnancy (beyond first trimester as the physiology of heartburn symptoms in pregnant women is different from that in the non-pregnant population), dementia, or uncontrolled psychiatric disease.

3. Subjects unable to complete a paper symptom diary for 6 of 7 days prior to their baseline visit.

4. Subjects whose symptoms are predominantly dyspeptic more than heartburn or reflux.

5. Subjects who have used homeopathy for GI symptoms or have received constitutional homeopathic treatment within the past 2 weeks.

6. Subjects taking herbal products or other supplements for GERD or dyspepsia related symptoms (includes peppermint oil).

7. Subjects who have taken > 12 doses of NSAIDS within the prior 30 days (aspirin ≤ 325 mg daily is allowed).

8. Subjects with lactose intolerance. Our rationale: the Acidil and placebo tablets both contain lactose. Lactose intolerance is associated with GI symptoms, some of which overlap with symptoms that we are measuring in this study. Excluding lactose interolerant individuals will help make our data more interpretable.

*If this population of subjects is too difficult to recruit, we will instead:

A) offer to enroll subjects if they wish to taper off their current medications. This will only be done if they do not have known Barrett's esophagus and are on a low dose PPI or H2 blocker. Subjects will be given standardized instructions for how to taper off of these medications using oral antacids as a bridge.

If we are unable to reach recruitment targets using secondary strategy A, we will also:

B) try to recruit subjects currently on stable drug therapy (no changes in PPI or H2 blocker dose x 2 weeks) with refractory GERD symptoms 3 or more days per week.

If we enroll subjects in groups A and/or B, we will modify our randomization approach to stratify by the different groups of subjects (i.e., based on status of PPI/H2 blocker use: none, recent, current) enrolled in the study so that they are equally distributed between arms.

B4. POSSIBLE BENEFITS

Study subjects may experience improvement in their GERD and/or dyspeptic symptoms by participating in this study. The placebo response in GERD-related trials may be as high as 40% (Cremonini). Participants will also be remunerated for their time. More generally, this study will provide preliminary effect size estimates and may further our understanding of the effects of this supplement (Acidil) and elements that contribute to the placebo response in GERD.

B5. POSSIBLE RISKS AND ANALYSIS OF RISK/BENEFIT RATIO

Homeopathic medicines are generally regarded as relatively safe due to the low concentrations of active ingredients present. In the case of Acidil, all of the included substances are at a 4C potency, the equivalent of a 10^{-8} dilution from the original starting concentration. Per Boiron, the manufacturer of Acidil, in reviewing their pharmacovigilence data, there have been no adverse event reports for this product over the past 10 years in the United States.

Nonetheless, some potential risks to subjects are as follows:

a) Homeopathic practitioners describe a phenomenon known as "proving" in which a patient begins to experience worsening of symptoms after an initial improvement. This is thought to occur when a patient has received too much medication and the problem usually resolves when the patient stops taking the medicine or takes it less frequently.

b) Questionnaires about symptom frequency and severity and quality of life: behavioral data collection involves virtually no risk, however, psychosocial tests may cause minor emotional distress. Because participants are free to stop at any time and will be reminded of this throughout the assessment process, the risk of distress is low. In our experience, behavioral tests are well tolerated and complications are extremely rare.

c) Some subjects may experience distress knowing that they are being video or audio recorded. If this is the case, they may decline this portion of the study without being disqualified from further participation. The risk of breach of confidentiality of this data is low as all audio and video data will be collected and stored digitally on a password protected server.

d) Keeping all data in locked files and stored on BIDMC hard drives that are behind a secure firewall will minimize the risk of inadvertent release of participant data. Participant data will be stored in de-identified form and will be identified only by study ID number. Corresponding participant names and PHI will be kept by the PI in a separate locked file.

Overall, the potential risks to participants are modest and are likely to be viewed as reasonable in relation to the anticipated benefits to subjects and others.

Any adverse event will be reported to the BIDMC IRB. In addition, we will convene an independent

monitoring committee approximately half way through subject recruitment to review study safety. The committee will be comprised of two individuals who are not involved in the study and have no substantive conflicts of interest. If, after reviewing the data, they feel there is evidence suggesting potential harm that warrants early termination of the study, the study will be stopped.

B6. RECRUITMENT AND CONSENT PROCEDURES

Recruitment

Subjects will be recruited initially from BIDMC HCA and GI clinics, but may eventually be recruited from other locations in the city. Additional locations may include local health food stores, the Marino Center (an integrative medicine clinic with sites in Sommerville and Waltham), and BIDMC-affiliated community primary care clinics. Permission to recruit study participants will be obtained from each site individually. Initial recruitment efforts will rely upon posted flyers (Appendix E). If the response to posted flyers is too slow, we will ask physicians to consider sending letters (Appendix E) to patients who may be eligible to introduce the study. Letters will be signed by both the patient's physician and study PI.

Flyers and letters will direct potential study participants to call a dedicated study phone number if they wish to receive further information about the study. Interested individuals will undergo a brief telephone screen to assess eligibility as described in Appendix A. Study calls will be returned 10am-9pm Monday thru Friday and 10am-5pm Saturday and Sunday, unless the caller specifically requests a call back outside of this time. If deemed ineligible to participate in the study, individuals will be referred back to their physician if they are seeking further care. If eligible, they will be sent a baseline symptom diary and scheduled for an intake visit as described above.

Consent

Written informed consent will be obtained by the research assistant or study physician at the initial visit in the BIDMC GCRC after reviewing inclusion and exclusion criteria, the participant's symptom diary, and the online medical record (OMR) if the participant is a patient at BIDMC. Subjects will be asked to participate in a study examining the effect of a natural supplement containing a low dose combination of 4 herbs on heartburn and GERD-related symptoms. There will not be any specific mention of an intent to understand how the doctor-patient interaction or beliefs and expectations affect response to treatment. Informed consent will be obtained using the following process:

- The subject will be asked to review the study consent form;
- A member of the research staff will meet with the subject to review the form, to confirm the subject's understanding of the study, and to answer any questions that the subject might have; and
- Once the subject demonstrates understanding of the study and agrees to participate in the study, the consent will be signed by both the subject and research staff member.
- The consent form will allow subjects the opportunity to specifically decline audio and/or video recording of their interaction with the study physician.

Subject Protection

Subjects may withdraw from the trial at any time and they will be informed that the study is entirely voluntary and that their clinical care will not be affected by this action. We will not specifically seek to enroll specific vulnerable populations.

B7. STUDY LOCATION

Privacy

Initial screening for the study will occur by telephone and will require potential subjects to initiate the process by calling the study phone number and indicating an interest in participating in the study. This process will not require extensive discussion of sensitive information. Formal consent to participate will occur in the GCRC in a private setting. All anthropomorphic (e.g., height and weight) and questionnaire data gathered as part of the study as well as conversations with the study physician discussing symptoms will also occur in a private setting in the GCRC. In all cases, the information gathered will be limited only to the data necessary to accomplish the research purposes.

Physical Setting

All subject interviews and testing will occur in the BIDMC GCRC except for circumstances in which the subject cannot return for the follow-up visit and elects to complete the second set of questionnaires from home or over the telephone.

B8. DATA SECURITY

All written/questionnaire data will be stored in a locked file cabinet in the study physician's office, which itself is locked whenever no one is present in the office. The only identifier that will be present on this data will be a unique, subject-specific study ID. This data will also be transferred to the password-protected database. We plan to utilize the REDCap system sponsored through BIDMC and the Harvard Catalyst Program. In addition, all computer files will be password protected. The study database will not include data items that uniquely identify participants such as subject name, address, phone number, social security number and medical record number, but will be indexed by subjects' study IDs. All data will be processed in aggregate form. The master list linking subjects' study IDs to personally identifiable information will be on file in a locked cabinet in the study physician's office. Electronic communication with outside collaborators will only involve deidentified information.

B9 Multi-Site Studies

Is the BIDMC the coordinating site or is the BIDMC PI the lead investigator of the multi-site study?

☐ Yes ☒ No *Not applicable. This is not a multi-site study.*

References

1. Zhao Y, William Encinosa W. Gastroesophageal reflux disease (GERD) hospitalizations in 1998 and 2005. HCUP Statistical Brief. Rockville, MD: AHRQ; 2008 January 2008. Report No.: #44.
2. Vakil N. Prescribing proton pump inhibitors: Is it time to pause and rethink? Drugs. 2012 Mar 5;72(4):437-45.

3. Heidelbaugh JJ, Metz DC, Yang YX. Proton pump inhibitors: Are they overutilised in clinical practice and do they pose significant risk? Int J Clin Pract. 2012 Jun;66(6):582-91.
4. Fass R, Sifrim D. Management of heartburn not responding to proton pump inhibitors. Gut. 2009 Feb;58(2):295-309.
5. Cremonini F, Ziogas DC, Chang HY, Kokkotou E, Kelley JM, Conboy L, et al. Meta-analysis: The effects of placebo treatment on gastro-oesophageal reflux disease. Aliment Pharmacol Ther. 2010 Jul;32(1):29-42.
6. Finniss DG, Benedetti F. Mechanisms of the placebo response and their impact on clinical trials and clinical practice. Pain. 2005 Mar;114(1-2):3-6.
7. Kaptchuk TJ, Kelley JM, Conboy LA, Davis RB, Kerr CE, Jacobson EE, et al. Components of placebo effect: Randomised controlled trial in patients with irritable bowel syndrome. BMJ. 2008 May 3;336(7651):999-1003.
8. Hall KT, Lembo AJ, Kirsch I, Ziogas DC, Douaiher J, Jensen KB, et al. Catechol-O-methyltransferase val158met polymorphism predicts placebo effect in irritable bowel syndrome. PLoS One. 2012;7(10):e48135.
9. Barnes PM, Bloom B, Nahin RL. Complementary and alternative medicine use among adults and children: United states, 2007. Natl Health Stat Report. 2008 Dec 10;(12)(12):1-23.
10. Jonas WB, Levin J. Essentials of complementary and alternative medicine. Philadelphia: Lippincott Williams and Wilkins; 1999.
11. Matthiessen PF, Bornhoft G. Homeopathy in healthcare: Effectiveness, appropriateness, safety, costs. Springer; 2011.
12. Paterson C, Ewings P, Brazier JE, Britten N. Treating dyspepsia with acupuncture and homeopathy: Reflections on a pilot study by researchers, practitioners and participants. Complement Ther Med. 2003 Jun;11(2):78-84.
13. Bell IR, Lewis DA,2nd, Brooks AJ, Schwartz GE, Lewis SE, Walsh BT, et al. Improved clinical status in fibromyalgia patients treated with individualized homeopathic remedies versus placebo. Rheumatology (Oxford). 2004 May;43(5):577-82.
14. Endrizzi C, Rossi E, Crudeli L, Garibaldi D. Harm in homeopathy: Aggravations, adverse drug events or medication errors? Homeopathy. 2005 Oct;94(4):233-40.
15. Borneman JP, Field RI. Regulation of homeopathic drug products. Am J Health Syst Pharm. 2006 Jan 1;63(1):86-91.
16. Jacobs J, Herman P, Heron K, Olsen S, Vaughters L. Homeopathy for menopausal symptoms in breast cancer survivors: A preliminary randomized controlled trial. J Altern Complement Med. 2005 Feb;11(1):21-7.
17. Hyland ME, Lewith GT. Oscillatory effects in a homeopathic clinical trial: An explanation using complexity theory, and implications for clinical practice. Homeopathy. 2002 Jul;91(3):145-9.
18. Benedetti F, Maggi G, Lopiano L. Open versus hidden medical treatments: The patient's knowledge about a therapy affects the therapy outcome. Prevention & Treatment. 2003;6(1).
19. Code of Federal Regulations Title 45 Part 46.116d, (http://www.hhs.gov/ohrp/humansubjects/guidance/45cfr46.html#46.116, Revised 1/15/2009).
20. Acidil [Internet].: Boiron USA; 2012. Available from: http://www.boironusa.com/products/acidil-tablets/.
21. The homoeopathic pharmacopoeia of the united states [Internet].; 2012. Available from: http://www.hpus.com/.
22. The Committee on Pharmacopoeia of the American Institute of Homeopathy. The homoeopathic pharmacopoeia of the united states. 7th ed. ed. Philadelphia, PA: Boericke & Tafel; 1964.

23. Revicki DA, Wood M, Wiklund I, Crawley J. Reliability and validity of the gastrointestinal symptom rating scale in patients with gastroesophageal reflux disease. Qual Life Res. 1998 Jan;7(1):75-83.
24. Velanovich V. The development of the GERD-HRQL symptom severity instrument. Dis Esophagus. 2007;20(2):130-4.
25. Talley NJ, Verlinden M, Jones M. Quality of life in functional dyspepsia: Responsiveness of the nepean dyspepsia index and development of a new 10-item short form. Aliment Pharmacol Ther. 2001 Feb;15(2):207-16.
26. Ware J,Jr, Kosinski M, Keller SD. A 12-item short-form health survey: Construction of scales and preliminary tests of reliability and validity. Med Care. 1996 Mar;34(3):220-33.
27. Paterson C. Measuring outcomes in primary care: A patient generated measure, MYMOP, compared with the SF-36 health survey. BMJ. 1996 Apr 20;312(7037):1016-20.
28. Chey W, Huang B, Jackson RL. Lansoprazole and esomeprazole in symptomatic GERD: A doube-blind, randomized, multicentre trial in 3000 patients confirms comparable symptom relief. Clin Drug Invest. 2003;23(2):69-84.
29. Whale CA, MacLaran SJ, Whale CI, Barnett M. Pilot study to assess the credibility of acupuncture in acute exacerbations of chronic obstructive pulmonary disease. Acupunct Med. 2009 Mar;27(1):13-5.
30. Mercer SW, Maxwell M, Heaney D, Watt GC. The consultation and relational empathy (CARE) measure: Development and preliminary validation and reliability of an empathy-based consultation process measure. Fam Pract. 2004 Dec;21(6):699-705.
31. Bishop FL, Yardley L, Lewith G. Developing a measure of treatment beliefs: The complementary and alternative medicine beliefs inventory. Complement Ther Med. 2005 Jun;13(2):144-9.
32. Miner P,Jr, Orr W, Filippone J, Jokubaitis L, Sloan S. Rabeprazole in nonerosive gastroesophageal reflux disease: A randomized placebo-controlled trial. Am J Gastroenterol. 2002 Jun;97(6):1332-9.
33. Kahrilas PJ, Miner P, Johanson J, Mao L, Jokubaitis L, Sloan S. Efficacy of rabeprazole in the treatment of symptomatic gastroesophageal reflux disease. Dig Dis Sci. 2005 Nov;50(11):2009-18.
34. Fass R, Chey WD, Zakko SF, Andhivarothai N, Palmer RN, Perez MC, et al. Clinical trial: The effects of the proton pump inhibitor dexlansoprazole MR on daytime and nighttime heartburn in patients with non-erosive reflux disease. Aliment Pharmacol Ther. 2009 Jun 15;29(12):1261-72.
