# Supplementary material for: Patient-Provider Interactions Affect Symptoms in Gastroesophageal Reflux Disease: A Pilot Randomized, Double-Blind, Placebo-Controlled Trial
Source: PLoS One. 2015 Sep 30;10(9):e0136855. doi: 10.1371/journal.pone.0136855 (PMC4589338; doi:10.1371/journal.pone.0136855)
Supplement: S1 Scripts — (PDF) [file pone.0136855.s004.pdf]

Date: Study ID:

Standard Interview

Time Start: _________

Introduction:

Thank you for taking the time to fill out the surveys and for your willingness to participate in this research study. I am now going to spend some time asking you about your GI symptoms so that I can better understand them. I will also look at your answers to some of the survey questions and your symptom diary as we talk to make sure that I am getting the full picture.

1. First, tell me about when your GERD symptoms (heart burn, acid reflux) first began (how long ago) and how your symptoms have evolution over time

heartburn, acid reflux/regurgitation – started

frequency

severity

alleviating and exacerbating factors

2. Do you have any dyspeptic symptoms (bloating/fullness, belching/flatulence, pain, nausea, early satiety)?

3. Any other GI symptoms (look at GSRS, Nepean, & symptom diary)?

4. Presence of red flag symptoms (dysphagia, hemoptysis, abdominal mass)

Date: Study ID:

5. Have you had any tests to evaluate your symptoms?

6. Are there any lifestyle changes you've made or conventional medications or supplements or complementary and alternative treatments that you've tried?

[ ] Reviewed current medications and supplements

<u>Past Medical History:</u>

<u>Physical exam notes:</u>
General habitus:

Oropharynx:

Neck:

Abdomen:

Additional Notes:

Thank you again for your time and interest in the study. I think the study supplement will be helpful for your symptoms. I am going to step out of the room and will ask you to complete a couple of additional short surveys while I get the study medication from our research pharmacy. Please leave the surveys in the envelope when you are finished as I am not supposed to see them.

Time End: _________

Date: Study ID:

Expanded Interview

Time Start: _________

Introduction:

Thank you for taking the time to fill out the surveys and for your willingness to participate in this research study. I am now going to spend some time asking you about your GI symptoms so that I can better understand them. I will also look at your answers to some of the survey questions and your symptom diary as we talk to make sure that I am getting the full picture.

1. First, tell me about when your GERD symptoms (heart burn, acid reflux) first began (how long ago) and how your symptoms have evolution over time

heartburn, acid reflux/regurgitation – started

frequency

severity

alleviating and exacerbating factors (including foods)

2. Additional qualities of the GERD symptoms:

specific time of day or year when they are worse

nature of the reflux taste

sensation of heartburn pain

any co-occurring symptoms

3. Do you have any dyspeptic symptoms (bloating/fullness, belching/flatulence, pain, nausea, early satiety)?

Date: Study ID:

4. Additional qualities dyspeptic symptoms:
specific times when better/ worse

sensations & location

any co-occurring symptoms?

5. Any other GI symptoms (look at GSRS, Nepean, & symptom diary) & qualities?

6. Presence of red flag symptoms (dysphagia, hemoptysis, abdominal mass)

7. Have you had any tests to evaluate your symptoms?

8. Are there any lifestyle changes you've made or conventional medications or supplements or complementary and alternative treatments that you've tried?

[ ] Reviewed current medications and supplements

I am now going to ask you some questions about other symptoms you may be experiencing and aspects of your life experience that make you uniquely you. Some of the questions may seem a bit odd. The reason I am asking them is that the system of alternative medicine that the supplement comes from values tailoring treatments to the individual. We would like to see if there are ways to predict who might be a better responder to the supplement. If you feel uncomfortable answering any of the questions, you may decline to answer them. Okay?

Date: Study ID:

9. Past medical history? What other medical problems are you currently being treated for? Any active issues?

9. Do you have any additional symptoms that are ongoing that not GI-related? (Ask about location, timing, intensity, sensation, amel/agg factors (time, weather, other)

11. Tell me about your appetite and the foods that you crave and that you cannot stand.

12. How is your body temperature? Do you prefer to be warmer or cooler? Temperature effect on energy?

13. What kind of weather do you prefer?

14. Tell me about your sleep. Are you a restless sleeper? What position? Any recurring dreams?

15. Perspiration? y/n, odiferous? Color? Anything remarkable?

16. Women – ask about menstruation. Anything remarkable? Any effect on other symptoms?

17. Tell me about your temperament. How do you view yourself? Recurrent thoughts? What would your best friend/spouse say about you?

18. Any fears or phobias?

19. Stresses or traumas that affect symptoms?

Date: Study ID:

<u>Physical exam notes:</u>
General habitus:

Oropharynx:

Neck:

Abdomen:

Additional Notes:

Thank you again for your time and interest in the study. I think the study supplement will be helpful for your symptoms. I am going to step out of the room and will ask you to complete a couple of additional short surveys while I get the study medication from our research pharmacy. Please leave the surveys in the envelop when you are finished as I am not supposed to see them.

Time End: _________
